# Arab world’s growing contribution to global leishmaniasis research (1998–2017): a bibliometric study

**DOI:** 10.1186/s12889-019-6969-9

**Published:** 2019-05-22

**Authors:** Samah W. Al-Jabi

**Affiliations:** Department of Clinical and Community Pharmacy, College of Medicine and Health Sciences, An-Najah National University, Nablus, 44839 Palestine

**Keywords:** Leishmania, Leishmaniasis, Bibliometric, Scopus, Arab world

## Abstract

**Background:**

Leishmaniasis is a parasitic disease caused by a protozoan of the *Leishmania* genus, and is considered a neglected tropical disease. It still remains a main public health concern at global level and in Arab world mainly in low-income countries. Therefore, this study was designed to evaluate the Arab world’s growing contribution to global leishmaniasis research.

**Methods:**

This study describes a bibliometric review of all leishmaniasis research publications published between January 1998 and December 2017 indexed on the Scopus database.

**Results:**

The total number of publications published at global level was 17,570 papers, which achieves an average annual productivity of 878.50 papers publications. Brazil was responsible for the greatest output with the total number of publications of 3865 followed by the Unites States (*n* = 2729), India (*n* = 2119), the United Kingdom (*n* = 1363), and Spain (*n* = 1274). By limiting the analysis to the publications that have been published by Arab world, the research productivity was 993 papers, which represents 5.65% of total research output at global level in research regarding leishmaniasis. Tunisia was responsible for the greatest output from Arab world with the total number of publications of 297 followed by Sudan (*n* = 192), Saudi Arabia (*n* = 131), Morocco (*n* = 119) and Egypt (*n* = 67). Since 1998, the growth of publications on leishmaniasis fluctuates, overall showing a rising trend in both global and Arab world. There is a highly significant correlation between publication productivity related to leishmaniasis at global level and the Arab world (r = 0.936; *p*-value< 0.001). Leishmaniasis treatment, intracellular mechanism of infection, and lifecycle of *leishmania* are the major current hot topics for the research in this subject at global level and the Arab world.

**Conclusions:**

The current study presents a novel review of the current Arab leishmaniasis-related research, and how these results are related to worldwide output. In comparison to the global research output, the Arab world produced less leishmaniasis research. The data presented in the current study by this innovative approach may serve relevant researchers to direct the global leishmaniasis research to Arab counties in which leishmaniasis is endemic.

## Background

Leishmaniasis is a parasitic disease caused by a protozoan of the *Leishmania* genus, and is considered a neglected tropical disease [1]. It is transmitted by the bite of infected female phlebotomine sand flies to mammals, including human beings [2, 3]. The most common types of leishmaniasis are visceral leishmaniasis, cutaneous leishmaniasis, and mucocutaneous leishmaniasis [4]. According to World Health Organization (WHO) update (2017), leishmaniasis is the main parasitic killer responsible for an estimated one million new cases of leishmaniasis and 20,000 to 30,000 deaths annually [5].

The clinical manifestations of leishmaniasis can range from subclinical (inapparent), or a self-resolving cutaneous ulcer to a disseminated infection (cutaneous, mucosal, or visceral) and even to a lethal systemic illness [6–10]. *Leishmania* infects some of the poorest people in the world, and is linked to population displacement, malnutrition lack of financial resources, and poor housing [5]. Leishmaniasis is a public health problem and it is endemic in many parts of the tropics, subtropics and the Mediterranean [11, 12]. Middle Eastern countries including Arab world’s countries are currently considered to be at risk from leishmaniasis because these countries are endemic for visceral and cutaneous leishmaniasis, and a huge deal of human migration from neighbouring countries is observed [13–17].

To encourage and strengthen research capacity in the field of neglected tropical diseases, including leishmaniasis, the WHO highlights the periodic review and development of present and national research agendas [18]. A number of global bibliometric studies exist in various infectious diseases [19–22], including tropical medicine [23–26]. In addition, several studies have evaluated the research output for leishmaniasis [25, 27–30]. Previous reports about leishmaniasis have mainly assessed the research output of international studies, and paid less interest on leishmaniasis research structure in Arab world. In other words, there is a lack of bibliometric studies regarding leishmaniasis in Arab world that evaluates the research output in a qualitative and a quantitative way, and the relationship among research hot topics was not revealed obviously. Therefore, it is essential to evaluate the scientific research output of the Arab world relative to that worldwide. In particular, the current study aimed to analyze the contribution of the Arab scientific community with regard to global contribution in (i) leishmaniasis literature during the last two decades; (ii) international collaborative patterns; (iii) productivity of the most active institutions; (iv) productivity of the most relevant journals; (v) characteristics of highly cited papers; and (vi) hot research topics. The data in this study can present a clear picture on the research growth accomplished in the field of leishmaniasis research, and it can aid researchers and practitioners in recognizing fundamental influences of this field.

## Methods

All leishmaniasis research publications published between January 1998 and December 2017 indexed on the Scopus database were analysed while the ones published in 2018 were excluded because Scopus as a secondary source has not yet archived all the publications from the primary sources for this year. The Scopus was used because it is the most widely accepted and frequently used database for analysis of scientific publications in different fields [22, 31–34]. The search was completed in November 2018. A bibliometric filter to capture leishmaniasis related publications from the Scopus database was created by using the key words ‘leishmaniosis, or ‘Leishmaniasis’ or ‘leishmania’ or ‘kala-azar’ in the ‘title’ selection mode.

All documents referring to leishmaniasis research during the last two decades were assessed with the following aspects: document types, languages, yearly publications, countries and collaboration patterns, institutions, journals, h-index, citations, and research hotspots. The analysis focused on providing outputs for the top ten prolific of the following: countries, journals with their impact factors (IF), cited articles, and institutions as in the most previous bibliometric studies [19, 33, 35–37]. In the current study, IF for the most prolific journals were extracted according to the 2017 journal citation report (JCR) at the time of study. Based on downloaded publications from Scopus database, bibliometric maps were created to determine the hot topics using the VOSviewer software version 1.6.9 (freely available at www.vosviewer.com). The collected data from the Scopus were limited to all 22 Arab countries, including “Algeria, Bahrain, Comoros, Djibouti, Egypt, Iraq, Jordan, Kuwait, Lebanon, Libyan Arab Jamahiriya, Morocco, Mauritania, Oman, Palestine, Qatar, Syrian Arab Republic, Saudi Arabia, Sudan, Somalia, Tunisia, United Arab Emirates, and Yemen” [38].

### Statistical analysis

The Statistical Package for Social Sciences (SPSS) software version 16 was applied for analysis, while graphical research output was also conducted in Microsoft Excel. Pearson correlation coefficient was used to analyze trends in publication between publication productivity related to leishmaniasis at global level and productivity related to leishmaniasis from Arab world.

## Results

**Fig. 1:**
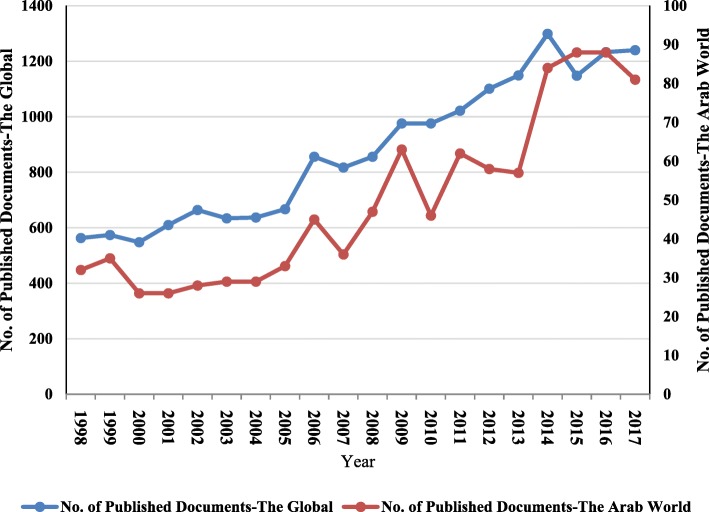
Publication distribution from worldwide and Arab world in leishmaniasis during 1998–2017

The total number of publications published between 1998 and 2017 at global level was 17,570 papers, which achieves an average annual productivity of 878.50 papers. The global research output consists of 15,021 articles (85.49%), followed by reviews (1175 papers, 6.69%), letters to the editor (615 papers, 3.50%). The remainders were other types (759 papers, 4.32%). Retrieved publications were written in 30 different languages, mainly English (*n* = 16,002; 91.08%) followed by Portuguese (*n* = 545; 3.10%), Spanish (*n* = 443; 2.52%), and French (*n* = 362; 2.06%). By limiting the analysis to the publications that have been published by Arab world, the research productivity was 993 papers during 1998–2017, which represents 5.65% of total scientific research output at global level in research related to leishmaniasis. The Arab world research output consists of 881 articles (88.72%), followed by reviews (51 papers, 5.14%), and letters to the editor (37 papers, 3.73%). The remainders were other types (24 papers, 2.42%). Retrieved publications from Arab world were written in 6 different languages, mainly English (*n* = 882; 88.82%). Since 1998, the growth of publications on leishmaniasis fluctuates, overall showing a rising trend in both global and Arab world (Fig. 1). There is a highly significant correlation between publication productivity related to leishmaniasis at global level and the Arab world (r = 0.936; *p*-value < 0.001).

A total of 135 countries worldwide contributed to the literature on leishmaniasis over the study period. The publications share of the top 10 most productive countries in leishmaniasis research ranges from 22.00% for Brazil to 3.35% for Canada during 1998–2017. Table 1 shows the top 10 top-ranking countries at global level in terms of relative contribution of each country to the total number of publications. Brazil was responsible for the greatest output with the total number of publications of 3865 followed by the Unites States (*n* = 2729), India (*n* = 2119), the United Kingdom (*n* = 1363) and Spain (*n* = 1274); (Table 1). The highest h-index value was 119 for the USA, followed by 102 for the UK, 83 for Brazil, 83 for India, and 82 for Germany. The USA, with the largest number of international collaboration publications (*n* = 1633), led this productivity rank followed by the UK (*n* = 937).

**Table 1:** Contribution and impact of the top 10 countries at global level in leishmaniasis research during 1998–2017

SCR	Country	Number of documents (%)	*h*-index	Number of collaborating countries	International collaborative publications
1st	Brazil	3865 (22.00)	83	64	910
2nd	USA	2729 (15.53)	119	100	1633
3rd	India	2119 (12.06)	83	68	558
4th	UK	1363 (7.76)	102	81	937
5th	Spain	1274 (7.25)	70	68	546
6th	Iran	1272 (7.24)	52	42	180
7th	France	1055 (6.00)	80	77	582
8th	Germany	839 (4.78)	82	68	522
9th	Italy	714 (4.06)	57	57	253
10th	Canada	588 (3.35)	75	55	341

*SCR* Standard competition ranking

Table 2 shows the ranking of Arab countries with relative contribution of each country to the total number of publications from Arab world. Tunisia was responsible for the greatest output with the total number of publications of 297 followed by Sudan (*n* = 192), Saudi Arabia (*n* = 131), Morocco (*n* = 119) and Egypt (*n* = 67); (Table 2). The highest h-index value for Arab countries was achieved by Sudan (43) followed by Tunisia (31), Morocco (21), Saudi Arabia (20), and Palestine (18). The Arab countries have collaborated with 52 countries/ territories in leishmaniasis research (Table 3).

**Table 2:** Ranking and contribution the Arab countries in leishmaniasis research during 1998–2017

SCR^a^	Country	Number of documents (%)	*h*-index	Number of collaborating countries	International collaborative publications
1st	Tunisia	297 (29.91)	31	38	125
2nd	Sudan	192 (19.34)	43	42	163
3rd	Saudi Arabia	131 (13.19)	20	36	92
4th	Morocco	119 (11.98)	21	24	45
5th	Egypt	67 (6.75)	15	29	55
6th	Algeria	61 (6.14)	14	24	45
7th	Iraq	44 (4.43)	9	6	10
8th	Palestine	40 (4.03)	18	23	40
9th	Syrian Arab Republic	37 (3.73)	11	23	15
10th	Lebanon	33 (3.32)	12	11	17
11th	Yemen	25 (2.52)	9	10	15
12th	Jordan	15 (1.51)	7	11	11
13th	Libyan Arab Jamahiriya	12 (12.1)	7	9	10
13th	Oman	12 (1.21)	6	3	6
15th	Bahrain	9 (0.91)	5	5	5
16th	Kuwait	6 (0.60)	5	4	3
17th	Qatar	5 (0.50)	3	5	5
18th	United Arab Emirates	4 (0.40)	2	2	2
19th	Somalia	1 (0.10)	1	2	1

*SCR* Standard competition ranking

^a^ Equal countries have the same ranking number, and then a gap is left in the ranking numbers

**Table 3:** Collaboration between Arab countries and non-Arab countries in leishmaniasis research during 1998–2017

Country	Number of documents	%	Country	Number of documents	%
France	132	13.29	Greece	6	0.60
United States	99	9.97	Nepal	6	0.60
United Kingdom	88	8.86	Colombia	5	0.50
Germany	51	5.14	Czech Republic	5	0.50
Belgium	47	4.73	Japan	5	0.50
Netherlands	43	4.33	Nigeria	4	0.40
Switzerland	39	3.93	Turkey	4	0.40
India	37	3.73	Cuba	3	0.30
Israel	29	2.92	Russian Federation	3	0.30
Spain	24	2.42	South Africa	3	0.30
Kenya	23	2.32	Austria	2	0.20
Brazil	20	2.01	Ghana	2	0.20
Ethiopia	17	1.71	Luxembourg	2	0.20
Pakistan	17	1.71	Malaysia	2	0.20
Italy	15	1.51	Uzbekistan	2	0.20
Canada	14	1.41	Venezuela	2	0.20
Sweden	14	1.41	Albania	1	0.10
Denmark	13	1.31	Burkina Faso	1	0.10
Uganda	12	1.21	Congo	1	0.10
Iran	10	1.01	Croatia	1	0.10
Australia	9	0.91	Ecuador	1	0.10
Malawi	9	0.91	Guatemala	1	0.10
Portugal	9	0.91	Hong Kong	1	0.10
Bangladesh	7	0.70	Mexico	1	0.10
Peru	7	0.70	Singapore	1	0.10
China	6	0.60	Thailand	1	0.10

Top 10 journals with the most publications at global level are presented in Table 4, representing 3505 publications accounting for 19.95% of the total publications. The most prolific journals in the field of leishmaniasis were *Plos Neglected Tropical Diseases* (*n* = 523, I.F. = 4.367), *American Journal of Tropical Medicine and Hygiene* (*n* = 492, I.F = 2.564), and *Experimental Parasitology* (*n* = 364, I.F. = 1.821). While Table 5 shows the top 10 journals with most of the publications from Arab world, representing 302 publications accounting for 30.41% of the total publications. The *Acta Tropica* published most of the publications (*n* = 46, I.F = 2.509), followed by *Transactions of the Royal Society of Tropical Medicine and Hygiene* (*n* = 44, I.F. = 2.820), and *Plos Neglected Tropical Diseases* (*n* = 42, I.F. = 4.367).

**Table 4:** Top 10 journals related to leishmaniasis research at global level during 1998–2017

SCR^a^	Journal	Number of documents (%)	IF^b^
1st	*Plos Neglected Tropical Diseases*	523 (2.98)	4.367
2nd	*American Journal of Tropical Medicine and Hygiene*	492 (2.80)	2.564
3rd	*Experimental Parasitology*	364 (2.07)	1.821
4th	*Acta Tropica*	354 (2.01)	2.509
5th	*Plos One*	321 (1.83)	2.766
6th	*Transactions of the Royal Society of Tropical Medicine and Hygiene*	307 (1.75)	2.820
7th	*Veterinary Parasitology*	291 (1.66)	2.422
8th	*Memorias do Instituto Oswaldo Cruz*	286 (1.63)	2.833
8th	*Revista da Sociedade Brasileira de Medicina Tropical*	286 (1.63)	1.358
10th	*Molecular and Biochemical Parasitology*	281 (1.60)	1.744

*SCR* Standard competition ranking; IF, Impact factor

^a^ Equal journals have the same ranking number, and then a gap is left in the ranking numbers

^b^ Impact factors (IF) based on Journal Citation Reports (JCR) 2017 from Clarivate Analytics

**Table 5:** Top 10 journals related to leishmaniasis research from Arab world during 1998–2017

SCR	Journal	Number of documents (%)	IF^a^
1st	*Acta Tropica*	46 (4.63)	2.509
2nd	*Transactions of the Royal Society of Tropical Medicine and Hygiene*	44 (4.43)	2.820
3rd	*Plos Neglected Tropical Diseases*	42 (4.23)	4.367
4th	*Parasites and Vectors*	33 (3.32)	3.163
5th	*American Journal of Tropical Medicine and Hygiene*	32 (3.22)	2.564
6th	*Plos One*	25 (2.52)	2.766
7th	*Bulletin de La Societe de Pathologie Exotique*	24 (2.42)	NA
8th	*Annals of Tropical Medicine and Parasitology* ^*b*^	20 (2.01)	1.703
9th	*Tropical Medicine and International Health*	19 (1.91)	2.541
10th	*Saudi Medical Journal*	17 (1.71)	1.055

*SCR* Standard competition ranking; IF, Impact factor; NA, not available

^a^ Impact factors (IF) based on Journal Citation Reports (JCR) 2017 from Clarivate Analytics

^b^ Currently known as: Pathogens and Global Health (2012 - current)

**Fig. 2:**
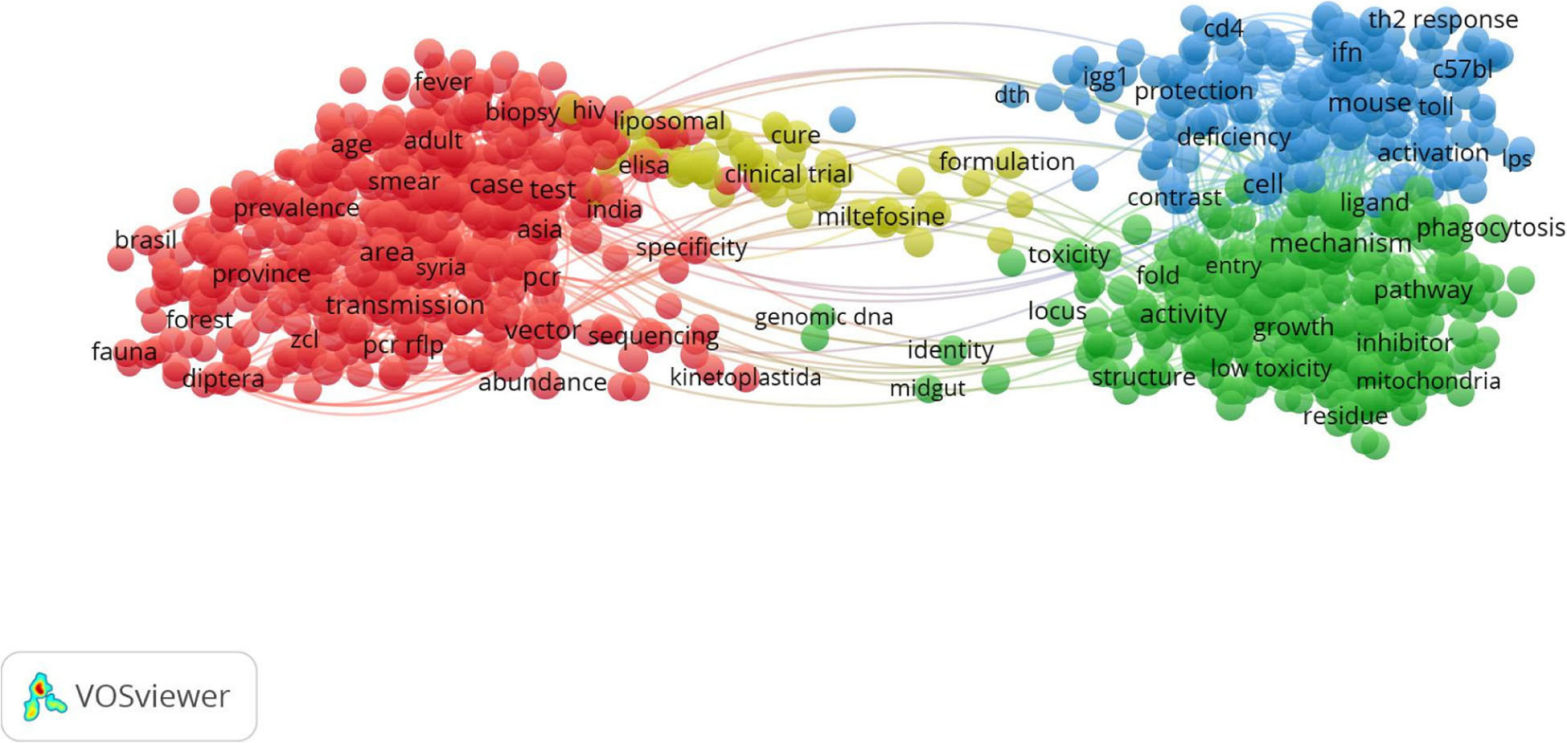
VOSviewer occurrence term map of the global hot topics of leishmaniasis research over the period of 1998–2017 which extracted from titles and abstracts of publications in the Scopus. The size and color a term indicate the frequency and the cluster with which the terms have been appeared respectively. In general, the closer two terms in the map indicating the stronger their relation. Out of 184,935 terms, 1586 terms meet the threshold by using minimum number of occurrence threshold of 50. By default, VOSviewer reducing the terms to the most relevant 60% results in 952 terms which encompass 4 main clusters in four colors: red, green, yellow, and blue

**Fig. 3:**
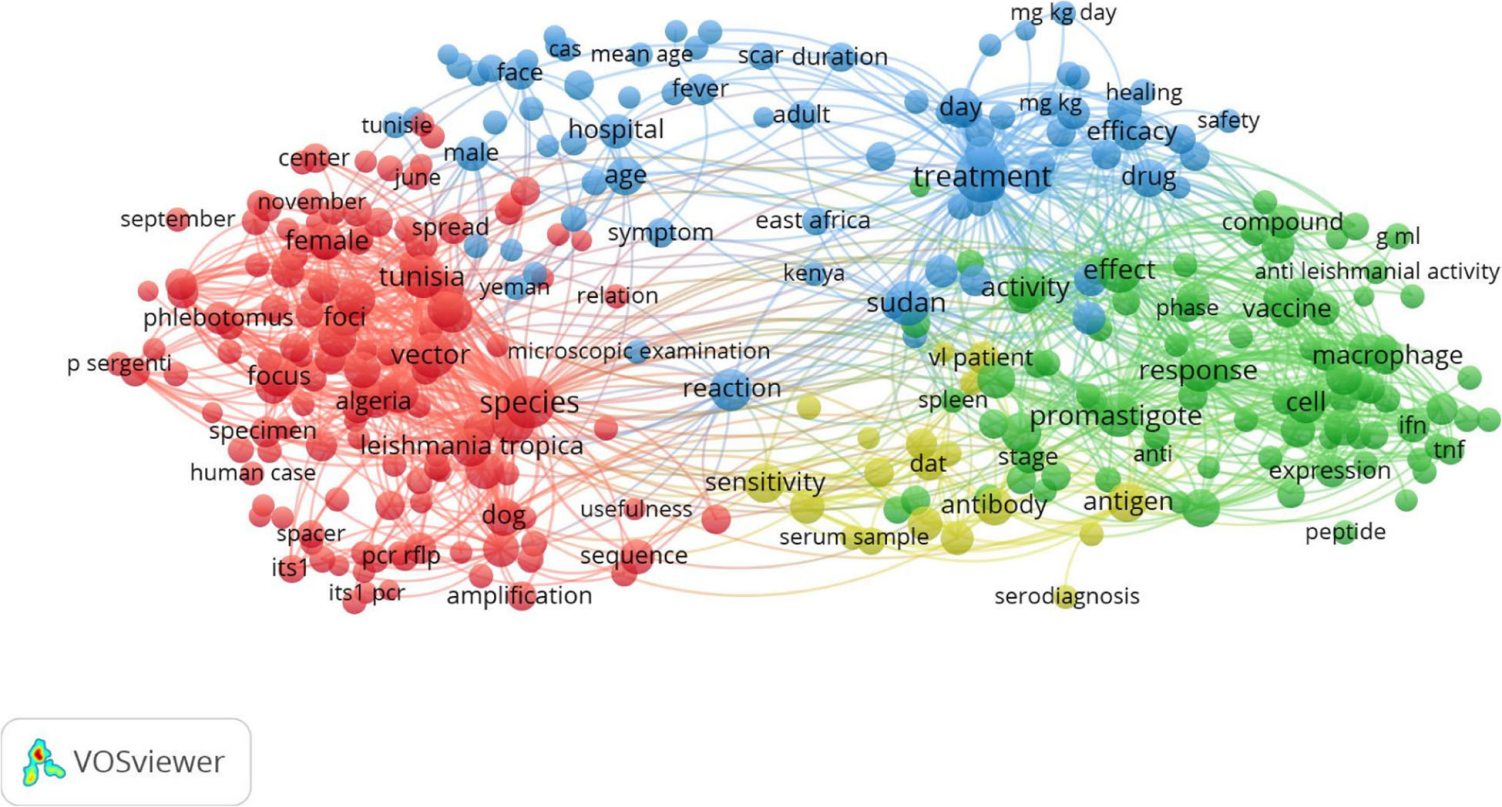
VOSviewer occurrence term map of the Arab world hot topics of leishmaniasis research over the period of 1998–2017 which extracted from titles and abstracts of publications in the Scopus. The size and color a term indicate the frequency and the cluster with which the terms have been appeared respectively. In general, the closer two terms in the map indicating the stronger their relation. Out of 17,479 terms, 531 terms meet the threshold by using minimum number of occurrence threshold of 10. By default, VOSviewer reducing the terms to the most relevant 60% results in 319 terms which encompass 4 main clusters in four colors: red, green, yellow, and blue

Figure 2 illustrates the term map of the global hot topics of leishmaniasis research over the period of 1998–2017 as extracted from titles and abstracts of publications. The term map was set up based on 952 terms encompass 4 main clusters in four colors: red, green, yellow, and blue. The red cluster included terms that were mainly related to the leishmaniasis causes and epidemiology research topics. The yellow cluster included terms closely related to treatment research topics. The green cluster included terms mainly related to the intracellular mechanism of infection. Blue cluster included terms roughly related to the lifecycle of leishmania. Whereas Fig. 3 illustrates the term map of the Arab world hot topics of leishmaniasis research over the period of 1998–2017 as extracted from titles and abstracts of publications. The term map was set up based on 319 terms encompass 4 main clusters in four colors: red, green, yellow, and blue. The red cluster included terms that were mainly related to the lifecycle of leishmania. Green cluster included terms roughly related to the intracellular mechanism of infection. Blue cluster included terms that were mainly related to the prevention, and treatment. The yellow cluster included terms closely related to the immunology of leishmaniasis.

The top 10 most cited publications [6, 39–47] at global level on leishmaniasis are presented in Table 6. From 1998 to 2017, the most frequently cited publication “Leishmaniasis: Current situation and new perspectives” published by *Comparative Immunology, Microbiology and Infectious Diseases* was from Switzerland by Desjeux [43] in 2004. The top 10 most cited publications [10, 48–56] from Arab world on leishmaniasis are presented in Table 7. From 1998 to 2017, the most frequently cited publication “Cutaneous leishmaniasis” published by *Lancet Infectious Diseases* was from Tunisia with international collaboration by Reithinger et al. [10] in 2007.

**Table 6:** Most 10 frequently cited publications on leishmaniasis research at global level during 1998–2017

SCR	Authors	Title	year of publication	Source title	Cited by
1st	Desjeux [43]	Leishmaniasis: Current situation and new perspectives	2004	*Comparative Immunology, Microbiology and Infectious Diseases*	1971
2nd	Alvar et al. [39]	Leishmaniasis worldwide and global estimates of its incidence	2012	*PLoS ONE*	1400
3rd	Belkaid et al. [40]	CD4 + CD25+ regulatory T cells control Leishmania major persistence and immunity	2002	*Nature*	1277
4th	Herwaldt [44]	Leishmaniasis	1999	*Lancet*	1204
5th	Murray et al. [6]	Advances in leishmaniasis	2005	*Lancet*	1079
6th	Croft et al. [41]	Drug resistance in leishmaniasis	2006	*Clinical Microbiology Reviews*	977
7th	Ivens et al. [45]	The genome of the kinetoplastid parasite, Leishmania major	2005	*Science*	912
8th	Darrah et al. [42]	Multifunctional TH1 cells define a correlate of vaccine-mediated protection against Leishmania major	2007	*Nature Medicine*	880
9th	Sacks et al. [46]	The immunology of susceptibility and resistance to Leishmania major in mice	2002	*Nature Reviews Immunology*	764
10th	Chappuis et al. [47]	Visceral leishmaniasis: What are the needs for diagnosis, treatment and control?	2007	Nature Reviews Microbiology	704

*SCR* Standard competition ranking

**Table 7:** Most 10 frequently cited publications on leishmaniasis research from Arab world during 1998–2017

SCR	Authors	Title	year of publication	Source title	Cited by
1st	Reithinger et al. [10]	Cutaneous leishmaniasis	2007	*Lancet Infectious Diseases*	685
2nd	Zijlstra et al. [55]	Post-kala-azar dermal leishmaniasis	2003	*Lancet Infectious Diseases*	282
3rd	Alrajhi et al. [48]	Fluconazole for the treatment of cutaneous leishmaniasis caused by Leishmania major	2002	*New England Journal of Medicine*	234
4th	El Tai et al. [51]	Genetic heterogeneity of ribosomal internal transcribed spacer in clinical samples of Leishmania donovani spotted on filter paper as revealed by single-strand conformation polymorphisms and sequencing	2000	*Transactions of the Royal Society of Tropical Medicine and Hygiene*	199
5th	Bacaër and Guernaoui [50]	The epidemic threshold of vector-borne diseases with seasonality: The case of cutaneous leishmaniasis in Chichaoua, Morocco	2006	*Journal of Mathematical Biology*	181
6th	Khalil et al. [52]	Autoclaved Leishmania major vaccine for prevention of visceral leishmaniasis: A randomised, double-blind, BCG-controlled trial in Sudan	2000	*Lancet*	173
7th	Arnoult et al. [49]	On the evolution of programmed cell death: Apoptosis of the unicellular eukaryote Leishmania major involves cysteine proteinase activation and mitochondrion permeabilization	2002	*Cell Death and Differentiation*	165
8th	Postigo [54]	Leishmaniasis in the World Health Organization Eastern Mediterranean Region	2010	*International Journal of Antimicrobial Agents*	148
9th	Pitta et al. [53]	IL-17 and IL-22 are associated with protection against human kala azar caused by Leishmania donovani	2009	*Journal of Clinical Investigation*	146
10th	Zijlstra et al. [56]	Diagnosing visceral leishmaniasis with the recombinant K39 strip test: Experience from the Sudan	2001	*Tropical Medicine and International Health*	140

*SCR* Standard competition ranking

Table 8 presents the performances of the top 10 most prolific institutes in the field of leishmaniasis between 1998 and 2017 at global level, representing 5498 publications accounting for 31.29% of the total publications. Five of these ten institutes were located in Brazil. The *Fundacao Oswaldo Cruz* is the largest contributor publishing 1427 publications on leishmaniasis. The *Universidade de Sao Paulo* in Brazil, the *Universidade Federal de Minas Gerais* in Brazil, the *Tehran University of Medical Sciences* in Iran, and *Universidade Federal do Rio de Janeiro* in Brazil, ranked second to fifth, contributing 769, 562, 503, and 485 publications, respectively. Table 9 presents the performances of the top 10 most productive institutes in the field of leishmaniasis between 1998 and 2017 from Arab world or from international collaboration, representing 640 publications accounting for 64.45% of the total publications. The *Institut Pasteur de Tunis* is the largest contributor publishing 178 publications on leishmaniasis. The *Khartoum University* in Sudan, and the *Institute of Endemic Diseases Sudan* in Sudan, ranked second and third, contributing 147, and 82 publications, respectively.

**Table 8:** Top 10 institutions most productive of research publications on leishmaniasis at global level during 1998–2017

SCR	Institute	Country	Number of documents (%)
1st	*Fundacao Oswaldo Cruz*	Brazil	1427 (8.12)
2nd	*Universidade de Sao Paulo*	Brazil	769 (4.38)
3rd	*Universidade Federal de Minas Gerais*	Brazil	562 (3.20)
4th	*Tehran University of Medical Sciences*	Iran	503 (2.86)
5th	*Universidade Federal do Rio de Janeiro*	Brazil	485 (2.76)
6th	*Indian Institute of Chemical Biology*	India	385 (2.19)
7th	*Banaras Hindu University*	India	371 (2.11)
8th	*London School of Hygiene & Tropical Medicine*	UK	345 (1.96)
9th	*Prins Leopold Instituut voor Tropische Geneeskunde*	Belgium	328 (1.87)
10th	*Universidade Federal da Bahia*	Brazil	323 (1.84)

*SCR* Standard competition ranking

**Table 9:** Top 10 institutions most productive of research publications on leishmaniasis from or collaborating with Arab world affiliations during 1998–2017

SCR ^a^	Institute	Country	Number of documents (%)
1st	*Institut Pasteur de Tunis*	Tunisia	178 (17.93)
2nd	*Khartoum University*	Sudan	147 (14.80)
3rd	*Institute of Endemic Diseases Sudan*	Sudan	82 (8.26)
4th	*Prins Leopold Instituut voor Tropische Geneeskunde*	Belgium	40 (4.03)
5th	*Charité – Universitätsmedizin Berlin*	Germany	38 (3.83)
6th	*University of Tunis El Manar*	Tunisia	35 (3.52)
7th	*Al-Quds University*	Palestine	31 (3.12)
8th	*Institut Pasteur - Alger*	Algeria	30 (3.02)
8th	*Universite de Montpellier*	France	30 (3.02)
10th	*Universite de Tunis El Manar, Hopital la Rabta*	Tunisia	29 (2.92)

*SCR* Standard competition ranking

^a^ Equal institutes have the same ranking number, and then a gap is left in the ranking numbers

## Discussion

This study has made a comprehensive research on scientific research output of the Arab world relative to that worldwide in the field of leishmaniasis. The research findings have indicated that leishmaniasis has attracted more and more attention from Arab and worldwide scholars over the past decade. However, despite a significant growth of leishmaniasis publications in Arab world and at global level, the distributions are highly unbalanced at some regional levels. As a result of the current bibliometric analysis, researchers can get basic information on leishmaniasis research such as hot research topics in a historic perspective.

However, from the Arab world only the top five countries -Tunisia, Sudan, Saudi Arabia, Morocco and Egypt - ranked well at global level as regards the number of publications related to leishmaniasis research: 17th, 24th, 32nd, 34th, and 40th, respectively. The current study shows that Arab countries are lagging behind most developed and developing countries in the number of publications related to leishmaniasis in contrast with high prevalence rate leishmaniasis in Arab countries. The WHO Eastern Mediterranean Region reported a very high proportion (82%) of countries endemic for cutaneous and visceral leishmaniasis [4]. The status of the health-research system in the Arab world has been described previously in numerous areas of health such as dengue research [21], pharmaceutical wastewater research [57], integrative and complementary medicine research [58], toxicology research [59], tobacco smoking research [60], breast cancer research [61], and infectious disease research [62]. Health-research systems in the Arab world are perceived as being non-productive system due to low priority in national research funding levels and development planning [63–65]. Despite the health services have improved in some Arab countries especially those with oil-based economies, the performance and development of their health-research system are lower than expected [66]. Generally, the amount of research related to medical field conducted in Arab world has grown considerably during the last decades and is still relatively small when compared with other world regions [61, 67–69].

Compared the current findings with the findings from developed countries, the Arab world produced less leishmaniasis research. This may be related to a relatively indigent economy in most Arab countries as reported in the online database of the World Bank [70]. In addition to a high poverty-growth elasticity for most Arab countries [71] according to population size and gross domestic product (GDP) per capita [70], which may lead to inadequate funding to support leishmaniasis research. For that reason, governments in the Arab world should give more attention to leishmaniasis research by offering more manpower and materials to support it. Also, the developed world should be persuaded to grant more collaborative plans with Arab world, and to attract more funding for leishmaniasis research and disease control.

Brazil is by far the most prolific country and is responsible for the greatest of number of publications in the field of leishmaniasis. A possible explanation for this finding may be due to high prevalence of leishmaniasis in this country which was exposed to many outbreaks [4, 72–75]. Additionally, other developing countries, such as India, and Iran, accounted in the most prolific countries in the field of the leishmaniasis research activity at global level, which may have been connected to a high prevalence of leishmaniasis in these countries [76–78].

In this study, Tunisia and Sudan had the highest research productivity in the field leishmaniasis. Previous bibliometric studies have assessed different issues in biomedical field in the Arab world [21, 59, 61, 66, 79–81]. Most of these studies found that Egypt and Saudi Arabia had the most research output among the Arab countries. No similar study has been found in a detailed literature search to address such those results but other related bibliometric studies have tried to make explanations for such findings [20–22, 35]. A possible explanation for these findings may be referring to leishmaniasis prevalence rates which are higher in Tunisia and Sudan. According to WHO report, zoonotic cutaneous leishmaniasis in Tunisia is endemic and considered a major public health problem and the annual incidence is approximately 30 per 100,000 people [82]. In Sudan, visceral leishmaniasis has been among the most important health problems [83]. Nearly 90% of global cases of visceral leishmaniasis occurred in the following countries: Brazil, Bangladesh, Ethiopia, India, and Sudan [4, 6]. Additionally, Afghanistan, Algeria, Brazil, Colombia, the Islamic Republic of Iran, Iraq, Morocco, Peru, Sudan, the Syrian Arab Republic, Tunisia and Yemen represent 90% of cutaneous leishmaniasis cases that are reported worldwide [4].

Leishmaniasis treatment, intracellular mechanism of infection, and lifecycle of *leishmania* are the major current hot topics for the research in this subject at global level and the Arab world. Furthermore, the major current hot topics in the current study are presented by the research highlighted in the most highly-cited publications [6, 10, 39–56], which gives an important and valuable insight into which publications and topics are motivating the research growth in this field over the time.

Similar to other studies [19, 33, 35–37], some limitations of this bibliometric study should be addressed. Although Scopus is one of the most largest global database [84], it might contain most publications in the field of leishmaniasis research. The main limitation relays to the citation and publications count applied for journals indexed by the Scopus. The citation and publication counts in these journals do not include citations and publications published in non-Scopus-indexed journals.

## Conclusions

The current study presents a novel review of the current Arab leishmaniasis-related research, and how these results are related to worldwide output. In summary, this study evaluated almost the last two decades of leishmaniasis literature output at the global level as well as the Arab world level. The findings of the current study indicated that Brazil was responsible for the greatest output in term of total number of publications in the field of leishmaniasis as indexed by Scopus during the period studied followed by the Unites States, India, the United Kingdom, and Spain. Additionally, Tunisia was responsible for the greatest output from Arab world followed by Sudan, Saudi Arabia, Morocco, and Egypt. In comparison to the global research output, the Arab world produced less leishmaniasis research. It can be concluded that research in the topics related to “leishmaniasis treatment”, “intracellular mechanism of infection”, and “lifecycle of *leishmania*” will undoubtedly continue to be the hotspots of leishmaniasis research at global level and the Arab world. In conclusion, the data presented in the current study by this innovative approach presents a clear picture on the research growth accomplished in the field of leishmania research, and may serve relevant researchers to direct the global leishmaniasis research as to Arab countries in which Leishmaniasis is endemic.

## Abbreviations

GDP: Gross domestic product
IFs: Impact factors
IQR: Interquartile range
JCR: Journal Citation Report
SPSS: Statistical Package for Social Sciences
WHO: World Health Organization

## References

[1] Ready PD (2014). Epidemiology of visceral leishmaniasis. Clin Epidemiol.

[2] Burza S, Croft SL, Boelaert M (2018). Leishmaniasis. Lancet..

[3] Oliveira F, Jochim RC, Valenzuela JG, Kamhawi S (2009). Sand flies, Leishmania, and transcriptome-borne solutions. Parasitol Int.

[4] World Health Organization (2017). Global leishmaniasis update, 2006-2015: a turning point in leishmaniasis surveillance. Wkly Epidemiol Rec.

[5] World Health Organization: Leishmaniasis: fact sheet [http://www.who.int/mediacentre/factsheets/fs375/en/]. Accessed 9 Jan 2018.

[6] Murray HW, Berman JD, Davies CR, Saravia NG (2005). Advances in leishmaniasis. Lancet.

[7] Alvar J, Canavate C, Gutierrez-Solar B, Jimenez M, Laguna F, Lopez-Velez R, Molina R, Moreno J (1997). Leishmania and human immunodeficiency virus coinfection: the first 10 years. Clin Microbiol Rev.

[8] Desjeux P (2001). The increase in risk factors for leishmaniasis worldwide. Trans R Soc Trop Med Hyg.

[9] Guerin PJ, Olliaro P, Sundar S, Boelaert M, Croft SL, Desjeux P, Wasunna MK, Bryceson AD (2002). Visceral leishmaniasis: current status of control, diagnosis, and treatment, and a proposed research and development agenda. Lancet Infect Dis.

[10] Reithinger R, Dujardin JC, Louzir H, Pirmez C, Alexander B, Brooker S (2007). Cutaneous leishmaniasis. Lancet Infect Dis.

[11] Oryan A, Akbari M (2016). Worldwide risk factors in leishmaniasis. Asian Pac J Trop Med.

[12] Di Muccio T, Scalone A, Bruno A, Marangi M, Grande R, Armignacco O, Gradoni L, Gramiccia M (2015). Epidemiology of imported Leishmaniasis in Italy: implications for a European endemic country. PLoS One.

[13] Jacobson RL (2011). Leishmaniasis in an era of conflict in the Middle East. Vector Borne Zoonotic Dis.

[14] Karunaweera ND, Ferreira MU (2018). Leishmaniasis: current challenges and prospects for elimination with special focus on the South Asian region. Parasitology.

[15] Salam N, Al-Shaqha WM, Azzi A (2014). Leishmaniasis in the middle east: incidence and epidemiology. PLoS Negl Trop Dis.

[16] Bahrami F, Harandi AM, Rafati S (2018). Biomarkers of Cutaneous Leishmaniasis. Front Cell Infect Microbiol..

[17] Gradoni L, Soteriadou K, Louzir H, Dakkak A, Toz SO, Jaffe C, Dedet JP, Campino L, Canavate C, Dujardin JC (2008). Drug regimens for visceral leishmaniasis in Mediterranean countries. Tropical Med Int Health.

[18] Hanney SR, Gonzalez-Block MA (2013). Organising health research systems as a key to improving health: the world health report 2013 and how to make further progress. Health Res Policy Syst.

[19] Sweileh WM, Al-Jabi SW, Sawalha AF, AbuTaha AS, Zyoud SH (2017). Bibliometric analysis of worldwide publications on antimalarial drug resistance (2006-2015). Malar Res Treat.

[20] Zyoud SH (2016). Global research trends of Middle East respiratory syndrome coronavirus: a bibliometric analysis. BMC Infect Dis.

[21] Zyoud SH (2016). Dengue research: a bibliometric analysis of worldwide and Arab publications during 1872-2015. Virol J.

[22] Zyoud SH (2017). Global toxocariasis research trends from 1932 to 2015: a bibliometric analysis. Health Res Policy Syst.

[23] Hagel C, Weidemann F, Gauch S, Edwards S, Tinnemann P (2017). Analysing published global Ebola virus disease research using social network analysis. PLoS Negl Trop Dis.

[24] Okorie PN, Bockarie MJ, Molyneux DH, Kelly-Hope LA (2014). Neglected tropical diseases: a systematic evaluation of research capacity in Nigeria. PLoS Negl Trop Dis.

[25] Perilla-Gonzalez Y, Gomez-Suta D, Delgado-Osorio N, Hurtado-Hurtado N, Baquero-Rodriguez JD, Lopez-Isaza AF, Lagos-Grisales GJ, Villegas S, Rodriguez-Morales AJ (2014). Study of the scientific production on leishmaniasis in Latin America. Recent Pat Antiinfect Drug Discov.

[26] Vera-Polania F, Perilla-Gonzalez Y, Martinez-Pulgarin DF, Baquero-Rodriguez JD, Munoz-Urbano M, Lagos-Gallego M, Lagos-Grisales GJ, Villegas S, Rodriguez-Morales AJ (2014). Bibliometric assessment of the Latin-American contributions in dengue. Recent Pat Antiinfect Drug Discov.

[27] Gonzalez-Alcaide G, Huamani C, Park J, Ramos JM (2013). Evolution of coauthorship networks: worldwide scientific production on leishmaniasis. Rev Soc Bras Med Trop.

[28] Ramos JM, Gonzalez-Alcaide G, Bolanos-Pizarro M (2013). Bibliometric analysis of leishmaniasis research in Medline (1945-2010). Parasit Vectors.

[29] Soosaraei M, Khasseh AA, Fakhar M, Hezarjaribi HZ (2018). A decade bibliometric analysis of global research on leishmaniasis in web of science database. Ann Med Surg (Lond).

[30] Huamani C, Romani F, Gonzalez-Alcaide G, Mejia MO, Ramos JM, Espinoza M, Cabezas C (2014). South American collaboration in scientific publications on leishmaniasis: bibliometric analysis in SCOPUS (2000-2011). Rev Inst Med Trop Sao Paulo.

[31] Zyoud SH (2018). Estimates of global research productivity in using nicotine replacement therapy for tobacco cessation: a bibliometric study. Glob Health.

[32] Cash-Gibson L, Rojas-Gualdron DF, Pericas JM, Benach J (2018). Inequalities in global health inequalities research: a 50-year bibliometric analysis (1966-2015). PLoS One.

[33] Al-Jabi SW (2017). Global trends in aspirin resistance-related research from 1990 to 2015: a bibliometric analysis. Basic Clin Pharmacol Toxicol.

[34] Singh N (2016). Scientometric analysis of research on Zika virus. Virusdisease.

[35] Al-Jabi SW (2017). Global research trends in West Nile virus from 1943 to 2016: a bibliometric analysis. Glob Health.

[36] Sweileh WM, Al-Jabi SW, AbuTaha AS, Zyoud SH, Anayah FMA, Sawalha AF (2017). Bibliometric analysis of worldwide scientific literature in mobile - health: 2006-2016. BMC Med Inform Decis Mak.

[37] Sweileh WM, Al-Jabi SW, Zyoud SH, Sawalha AF. Bibliometric analysis of literature in pharmacy education: 2000-2016. Int J Pharm Pract. 2018. 10.1111/ijpp.12429 29315940

[38] Nour SSOM (2013). The incidence and transfer of knowledge within the Arab societies. J Knowl Econ.

[39] Alvar Jorge, Vélez Iván D., Bern Caryn, Herrero Mercé, Desjeux Philippe, Cano Jorge, Jannin Jean, Boer Margriet den (2012). Leishmaniasis Worldwide and Global Estimates of Its Incidence. PLoS ONE.

[40] Belkaid Y, Piccirillo CA, Mendez S, Shevach EM, Sacks DL (2002). CD4+CD25+ regulatory T cells control Leishmania major persistence and immunity. Nature.

[41] Croft SL, Sundar S, Fairlamb AH (2006). Drug resistance in leishmaniasis. Clin Microbiol Rev.

[42] Darrah PA, Patel DT, De Luca PM, Lindsay RW, Davey DF, Flynn BJ, Hoff ST, Andersen P, Reed SG, Morris SL (2007). Multifunctional TH1 cells define a correlate of vaccine-mediated protection against Leishmania major. Nat Med.

[43] Desjeux P (2004). Leishmaniasis: current situation and new perspectives. Comp Immunol Microbiol Infect Dis.

[44] Herwaldt BL (1999). Leishmaniasis. Lancet.

[45] Ivens AC, Peacock CS, Worthey EA, Murphy L, Aggarwal G, Berriman M, Sisk E, Rajandream MA, Adlem E, Aert R (2005). The genome of the kinetoplastid parasite, Leishmania major. Science.

[46] Sacks D, Noben-Trauth N (2002). The immunology of susceptibility and resistance to Leishmania major in mice. Nat Rev Immunol.

[47] Chappuis F, Sundar S, Hailu A, Ghalib H, Rijal S, Peeling RW, Alvar J, Boelaert M (2007). Visceral leishmaniasis: what are the needs for diagnosis, treatment and control?. Nat Rev Microbiol.

[48] Alrajhi AA, Ibrahim EA, De Vol EB, Khairat M, Faris RM, Maguire JH (2002). Fluconazole for the treatment of cutaneous leishmaniasis caused by Leishmania major. N Engl J Med.

[49] Arnoult D, Akarid K, Grodet A, Petit PX, Estaquier J, Ameisen JC (2002). On the evolution of programmed cell death: apoptosis of the unicellular eukaryote Leishmania major involves cysteine proteinase activation and mitochondrion permeabilization. Cell Death Differ.

[50] Bacaer N, Guernaoui S (2006). The epidemic threshold of vector-borne diseases with seasonality: the case of cutaneous leishmaniasis in Chichaoua, Morocco. J Math Biol.

[51] El Tai NO, El Fari M, Presber W, Schönian G, Osman OF (2000). Genetic heterogeneity of ribosomal internal transcribed spacer in clinical samples of Leishmania donovani spotted on filter paper as revealed by single-strand conformation polymorphisms and sequencing. Trans R Soc Trop Med Hyg.

[52] Khalil EA, El Hassan AM, Zijlstra EE, Mukhtar MM, Ghalib HW, Musa B, Ibrahim ME, Kamil AA, Elsheikh M, Babiker A (2000). Autoclaved Leishmania major vaccine for prevention of visceral leishmaniasis: a randomised, double-blind, BCG-controlled trial in Sudan. Lancet.

[53] Pitta MG, Romano A, Cabantous S, Henri S, Hammad A, Kouriba B, Argiro L, el Kheir M, Bucheton B, Mary C (2009). IL-17 and IL-22 are associated with protection against human kala azar caused by Leishmania donovani. J Clin Invest.

[54] Postigo JA (2010). Leishmaniasis in the World Health Organization eastern Mediterranean region. Int J Antimicrob Agents.

[55] Zijlstra EE, Musa AM, Khalil EA, el-Hassan IM, el-Hassan AM (2003). Post-kala-azar dermal leishmaniasis. Lancet Infect Dis.

[56] Zijlstra EE, Nur Y, Desjeux P, Khalil EA, El-Hassan AM, Groen J (2001). Diagnosing visceral leishmaniasis with the recombinant K39 strip test: experience from the Sudan. Tropical Med Int Health.

[57] Zyoud SH, Zyoud SH, Al-Jabi SW, Sweileh WM, Awang R (2016). Contribution of Arab countries to pharmaceutical wastewater literature: a bibliometric and comparative analysis of research output. Ann Occup Environ Med.

[58] Zyoud SH, Al-Jabi SW, Sweileh WM (2015). Scientific publications from Arab world in leading journals of integrative and complementary medicine: a bibliometric analysis. BMC Complement Altern Med.

[59] Zyoud SH, Al-Jabi SW, Sweileh WM, Awang R (2014). A bibliometric analysis of toxicology research productivity in middle eastern Arab countries during a 10-year period (2003-2012). Health Res Policy Syst.

[60] Zyoud SH, Al-Jabi SW, Sweileh WM, Awang R (2014). A Scopus-based examination of tobacco use publications in middle eastern Arab countries during the period 2003-2012. Harm Reduct J.

[61] Sweileh WM, Zyoud SH, Al-Jabi SW, Sawalha AF (2015). Contribution of Arab countries to breast cancer research: comparison with non-Arab middle eastern countries. BMC Womens Health.

[62] Sweileh WM, Al-Jabi SW, Abuzanat A, Sawalha AF, AbuTaha AS, Ghanim MA, Zyoud SH (2015). Assessment of research productivity of Arab countries in the field of infectious diseases using web of science database. Infect Dis Poverty.

[63] El-Azami-El-Idrissi M, Lakhdar-Idrissi M, Ouldim K, Bono W, Amarti-Riffi A, Hida M, Nejjari C (2013). Improving medical research in the Arab world. Lancet.

[64] Sibai AM, Singh NV, Jabbour S, Saleh S, Abdulrahim S, Naja F, Yazbek S (2017). Does published research on non-communicable disease (NCD) in Arab countries reflect NCD disease burden?. PLoS One.

[65] Nakkash R, Afifi R, Maziak W (2014). Research and activism for tobacco control in the Arab world. Lancet.

[66] Benamer HT, Bakoush O (2009). Arab nations lagging behind other middle eastern countries in biomedical research: a comparative study. BMC Med Res Methodol.

[67] Saquib N, Zaghloul MS, Mazrou A, Saquib J (2017). Cardiovascular disease research in Saudi Arabia: a bibliometric analysis. Scientometrics.

[68] Al-Kindi S, Al-Juhaishi T, Haddad F, Taheri S, Abi Khalil C (2015). Cardiovascular disease research activity in the Middle East: a bibliometric analysis. Ther Adv Cardiovasc Dis.

[69] Jones AC, Geneau R (2012). Assessing research activity on priority interventions for non-communicable disease prevention in low- and middle-income countries: a bibliometric analysis. Glob Health Action.

[70] World bank group: countries and economies [https://data.worldbank.org/country]. Accessed 25 Nov 2018.

[71] Abu-Ismail K, Taleb GA, Ramadan R. Towards more sensible poverty measurement [http://www.arabstates.undp.org/content/rbas/en/home/library/Sustainable_development/the-adcr-2011-towards-more-sensible-poverty-measurement-.html]. Accessed 24 Nov 2018.

[72] Jeronimo SM, Duggal P, Braz RF, Cheng C, Monteiro GR, Nascimento ET, Martins DR, Karplus TM, Ximenes MF, Oliveira CC (2004). An emerging peri-urban pattern of infection with Leishmania chagasi, the protozoan causing visceral leishmaniasis in Northeast Brazil. Scand J Infect Dis.

[73] Jeronimo SM, Oliveira RM, Mackay S, Costa RM, Sweet J, Nascimento ET, Luz KG, Fernandes MZ, Jernigan J, Pearson RD (1994). An urban outbreak of visceral leishmaniasis in Natal, Brazil. Trans R Soc Trop Med Hyg.

[74] Silva ES, Gontijo CM, Pacheco RS, Fiuza VO, Brazil RP (2001). Visceral leishmaniasis in the metropolitan region of Belo Horizonte, state of Minas Gerais, Brazil. Mem Inst Oswaldo Cruz.

[75] Werneck GL, Rodrigues L, Santos MV, Araujo IB, Moura LS, Lima SS, Gomes RB, Maguire JH, Costa CH (2002). The burden of Leishmania chagasi infection during an urban outbreak of visceral leishmaniasis in Brazil. Acta Trop.

[76] Muniaraj M (2014). The lost hope of elimination of kala-azar (visceral leishmaniasis) by 2010 and cyclic occurrence of its outbreak in India, blame falls on vector control practices or co-infection with human immunodeficiency virus or therapeutic modalities?. Trop Parasitol.

[77] Fakoorziba MR, Baseri A, Eghbal F, Rezaee S, Azizi K, Moemenbellah-Fard MD (2011). Post-earthquake outbreak of cutaneous leishmaniasis in a rural region of southern Iran. Ann Trop Med Parasitol.

[78] Nateghi Rostami M, Saghafipour A, Vesali E (2013). A newly emerged cutaneous leishmaniasis focus in Central Iran. Int J Infect Dis.

[79] Bayoumy K, MacDonald R, Dargham SR, Arayssi T (2016). Bibliometric analysis of rheumatology research in the Arab countries. BMC Res Notes.

[80] Sweileh WM, Zyoud SH, Al-Jabi SW, Sawalha AF (2014). Quantity and quality of obesity-related research in Arab countries: assessment and comparative analysis. Health Res Policy Syst.

[81] Tadmouri GO, Bissar-Tadmouri N (2003). Biomedical publications in an unstable region: the Arab world, 1988-2002. Lancet.

[82] World Health Organization. Neglected tropical diseases: cutaneous leishmaniasis in Tunisia [http://www.emro.who.int/neglected-tropical-diseases/countries/cl-tunisia.html]. Accessed 26 Jan 2018.

[83] Zijlstra EE, el-Hassan AM (2001). Leishmaniasis in Sudan. Visceral leishmaniasis. Trans R Soc Trop Med Hyg.

[84] Falagas ME, Pitsouni EI, Malietzis GA, Pappas G (2008). Comparison of PubMed, Scopus, web of science, and Google scholar: strengths and weaknesses. FASEB J.

